# *LIN28B* gene polymorphisms modify hepatoblastoma susceptibility in Chinese children

**DOI:** 10.7150/jca.42798

**Published:** 2020-03-15

**Authors:** Zhonghua Yang, Yuyao Deng, Keren Zhang, Yuzuo Bai, Jinhong Zhu, Jiao Zhang, Yijuan Xin, Li Li, Jing He, Weilin Wang

**Affiliations:** 1 Department of Pediatric Surgery, Shengjing Hospital of China Medical University, Shenyang 110004, Liaoning, China.; 2 Department of Clinical Medicine, The Fourth Affiliated Hospital of China Medical University, Shenyang 110001, Liaoning, China.; 3 Department of Clinical Laboratory, Biobank, Harbin Medical University Cancer Hospital, Harbin 150040, Heilongjiang, China.; 4 Department of Pediatric Surgery, the First Affiliated Hospital of Zhengzhou University, Zhengzhou 450052, Henan, China.; 5 Clinical Laboratory Medicine Center of PLA, Xijing Hospital, Air Force Medical University, Xi'an 710032, Shaanxi, China.; 6 Kunming Key Laboratory of Children Infection and Immunity, Yunnan Key Laboratory of Children's Major Disease Research, Yunnan Institute of Pediatrics Research, Yunnan Medical Center for Pediatric Diseases, Kunming Children's Hospital, Kunming 650228, Yunnan, China.; 7 Department of Pediatric Surgery, Guangzhou Institute of Pediatrics, Guangdong Provincial Key Laboratory of Research in Structural Birth Defect Disease, Guangzhou Women and Children's Medical Center, Guangzhou Medical University, Guangzhou 510623, Guangdong, China.

**Keywords:** *LIN28B*, polymorphism, hepatoblastoma, susceptibility, case-control study

## Abstract

Hepatoblastoma is one of the malignant liver tumors in children. However, genetic mechanisms underpinning the initiation of hepatoblastoma remain largely unclear. The previous study showed that lin-28 homolog B (LIN28B) might play a role in the development of hepatoblastoma. To detect the association between *LIN28B* gene polymorphisms and hepatoblastoma risk in Chinese children, we conducted a five-center case-control study of 275 hepatoblastoma patients and 1018 cancer-free controls. Four potentially functional polymorphisms were genotyped using the Taqman method. Odds ratios (ORs) and 95% confidence intervals (CIs) were used to evaluate the strength of the associations. We found that the rs314276 C>A polymorphism (AA vs. CC: adjusted OR=2.05, 95% CI=1.36-3.10, *P*=0.0006; AA vs. CA/CC: adjusted OR=2.11, 95% CI=1.43-3.12, *P*=0.0002) and rs9404590 T>G (GG vs. TT: adjusted OR=1.89, 95% CI=1.20-3.00, *P*=0.007; GG vs. TT/TG: adjusted OR=1.87, 95% CI=1.20-2.92, *P*=0.006) were associated with increased hepatoblastoma risk. Combination analysis of risk genotypes showed that patients with four risk genotypes had a higher chance of developing hepatoblastoma than carriers of 1 to 3 risk genotypes. Stratification analysis showed the significant association between the rs314276 AA genotype and hepatoblastoma risk in both age and sex groups, as well as clinical stages III+IV cases. The rs9404590 GG genotype was associated with hepatoblastoma risk in participants' ≥17 months, in females, and for those with clinical stages III+IV disease. Furthermore, four risk genotypes confer higher hepatoblastoma susceptibility in both age and sex groups, as well as groups with clinical stages III+IV disease. Genotype-based gene expression analysis confirmed that the rs9404590 T>G polymorphism was significantly associated with altered *LIN28B* gene expression. We further validated our findings using false-positive probability analysis. This finding suggested that *LIN28B* gene polymorphisms may be associated with an increased predisposition to hepatoblastoma.

## Introduction

Hepatoblastoma is one of the major malignant tumors in children and originates from the progenitor cell during embryogenesis, accounting for two-thirds of the pediatric malignant tumors 1. The incidence rate is approximately 1.5 cases/million population per year (around 1% of cancers for young children) 2 and has been rising 4% per year continually 3. This phenomenon is partly due to increased prematurity and very low birth weight 4,5. The development of hepatoblastoma is also associated with some environmental factors, such as parental exposure to tobacco, metal and petroleum products 6-9. Moreover, it is strongly associated with familial adenomatous polyposis 10, Beckwith-Wiedemann syndrome 11, and Glycogen storage disease 12, indicating some genetic underpinnings. Although progressive surgical techniques and chemotherapy regimens have increased survival rates, the prognosis of high-risk hepatoblastoma remains very poor 13. As for the molecular mechanism, although Wnt/β-catenin, MYC, and Hippo pathways have been reported to be involved in the pathogenesis of hepatoblastoma 14-16, little is known about the molecular basis of this disease, and there are no validated prognostic or therapeutic biomarkers for hepatoblastoma patients.

*LIN28B* gene encodes an RNA-binding protein, which is featured by the conservative N-terminal cold-shock domain and two C-terminal CysCysHisCys zinc fingers 17. *LIN28B* functions by blocking the maturity of tumor-suppressing microRNA (miRNA) *let-7* family, which subsequently causes overexpression of numerous oncogenes, such as *C-MYC* and *K-RAS*, thereby supporting tumorigenesis and tumor growth 18,19. Meanwhile, *LIN28B* itself is downregulated by let-7, leading to the formation of double-negative feedback 20. In this way, *LIN28B* is aberrantly expressed in a broad spectrum of tumors and engaged in the regulation of miRNAs 18,20,21. A study showed that *LIN28B* is sufficient to drive liver tumors in the *let-7* miRNA dependent and independent ways in endogenous tumor models and is over-activated in mouse models of *MYC*-driven hepatoblastoma 19, which indicates its essential role in the development of hepatoblastoma.

The elucidation of genetic mechanisms of hepatoblastoma may accelerate the development of preventive oncology. Genome-wide association study (GWAS) is one of the emerging and promising techniques to associate genetic variations with disease risk. Most of the genetic variants are single nucleotide polymorphisms (SNPs). *LIN28B* polymorphisms were associated with Wilms tumor 22 and neuroblastoma 23 susceptibility in Chinese children. However, the relationship of *LIN28B* polymorphisms with hepatoblastoma susceptibility has not been investigated. In this study, we did a five-center case-control study to investigate the association between *LIN28B* gene polymorphisms and hepatoblastoma susceptibility in Chinese Han children.

## Material and methods

### Patients and controls

We enrolled 275 histopathologically diagnosed hepatoblastoma patients and 1018 cancer-free controls from Guangdong, Henan, Shaanxi, Yunnan and Liaoning provinces (**Supplemental Table 1**). All controls are unrelated to patients genetically. Moreover, controls were matched to patients by age, gender, and ethnicity. Our study was approved by the Ethics Committee of Guangzhou Women and Children's Medical Center. Written informed consent was obtained from each patient or his/her guardian. The study protocol was compliant with ethical guidelines.

### SNP selection and genotyping

Four *LIN28B* polymorphisms (rs314276 C>A, rs221634 A>T, rs221635 T>C and rs9404590 T>G) were chosen and genotyped using the TaqMan real-time PCR method as we reported previously 22, 23. Briefly, the selected polymorphisms were all potentially functional SNPs according to SNPinfo online software (https://snpinfo.niehs.nih.gov/snpinfo/snpfunc.html), which can affect the binding capacity of transcription factor binding sites (rs314276) or microRNA binding sites (rs221634 and rs221635), or leading to amino acids alterations (rs9404590). To validate the accuracy of genotyping results and for quality control, approximately 10% of the samples were randomly selected and re-genotyped. The concordance for the quality control samples was 100%.

### Genotype and gene expression correlation analysis

GTEx Portal database (https://www.gtexportal.org/home/) was used to evaluate the correlation between genotypes of the selected polymorphisms and *LIN28B* mRNA expression levels 24.

### Statistical analysis

The χ^2^ test was used to evaluate the demographic variables distribution, risk factors distribution, and *LIN28B* genotype distributions between case and control groups. The χ^2^ test was also performed to assess whether or not the *LIN28B* genotypes were consistent with Hardy-Weinberg equilibrium (HWE). Unconditional univariate and multivariate logistic regression analyses were used to estimate the strength of association between the selected polymorphisms and hepatoblastoma risk, using odds ratio (ORs) and 95% confidence intervals (CIs). Age and gender were adjusted for in the multivariate analysis. Further stratification analysis was performed based on the age, sex, and clinical stages. Moreover, we also performed false-positive probability analysis (FPRP) analysis to verify the significant results from the combined subjects 25. Differences with *P* values <0.05 were counted as statistically significant. All two-sided statistical analyses were performed using SAS software (version 9.1; SAS Institute, Cary, NC, United States).

## Results

### General characteristics of the subjects

As shown in **Supplemental Table 1**, there is no significant difference in both cases and controls in terms of age (*P*=0.365) and gender (*P*=0.589). The majority of the subjects in both cases and controls are male, accounting for 58.91% (162/275) and 60.71% (618/1018), respectively.

### Association of *LIN28B* SNPs with hepatoblastoma susceptibility

Of the included subjects, 275 cases and 1017 controls were successfully genotyped. The *LIN28B* genotypes are in accordance with HWE in the controls (*P*=0.209 for rs314276 C>A, *P*=0.969 for rs221634 A>T, *P*=0.139 for rs221635 T>C and *P*=0.868 for rs9404590 T>G). The genotype frequencies of four SNPs in cases and controls were listed in **Table 1**. Our results indicated that patients with the rs314276 A allele had higher cancer risk (AA vs. CC: adjusted OR=2.05, 95% CI=1.36-3.10, *P*=0.0006; AA vs. CA/CC: adjusted OR=2.11, 95% CI=1.43-3.12, *P*=0.0002). Carriers of the rs9404590 G allele also showed significantly increased risk (GG vs. TT: adjusted OR=1.89, 95% CI=1.20-3.00, *P*=0.007; GG vs. TT/TG: adjusted OR=1.87, 95% CI=1.20-2.92, *P*=0.006), compared with the reference group. According to the ORs, risk genotypes were carriers with rs314276 AA, rs221634 AA/AT, rs221635 TC/TT, rs9404590 TG/GG. Meanwhile, patients carrying four risk genotypes had a significantly elevated risk (adjusted OR=1.95, 95% CI=1.31-2.90, *P*=0.0009) when compared with those with 1-3 risk genotypes. Unfortunately, no significant association was detected for either rs221634 A>T or rs221635 T>C in any comparison.

### Stratification analysis

As shown in **Table 2**, we found a more prominent association for rs314276 AA genotype in the following subgroups: patients <17 months (adjusted OR=2.19, 95% CI=1.28-3.76, *P*=0.005) and ≥17 months (adjusted OR=2.04, 95% CI=1.16-3.58, *P*=0.013), females (adjusted OR=2.21, 95% CI=1.20-4.06, *P*=0.011) and males (adjusted OR=2.04, 95% CI=1.23-3.39, *P*=0.006), as well as patients with tumors in clinical stages III+IV (adjusted OR=3.15, 95% CI=1.73-5.73, *P*=0.0002). The association between the rs9404590 GG genotype and increased cancer risk was more pronounced in the strata of patients ≥17 months (adjusted OR=2.02, 95% CI=1.07-3.83, *P*=0.031), females (adjusted OR=2.09, 95% CI=1.02-4.28, *P*=0.044) and patients with tumor in III+IV clinical stages (adjusted OR=2.88, 95% CI=1.47-5.62, *P*=0.002). As for risk genotypes, the association between 4 risk genotypes and HB risk was statistically significant in patients younger (adjusted OR=1.88, 95% CI=1.08-3.29, *P*=0.027) and ≥17 months (adjusted OR=2.04, 95% CI=1.16-3.58, *P*=0.013), males (adjusted OR=1.94, 95% CI=1.03-3.63, *P*=0.039), and females (adjusted OR=1.95, 95% CI=1.17-3.25, *P*=0.010) and patients with tumor in III+IV clinical stages (adjusted OR=3.15, 95% CI=1.73-5.73, *P*=0.0002).

### Genotype-based mRNA expression analysis

**Figure 1:**
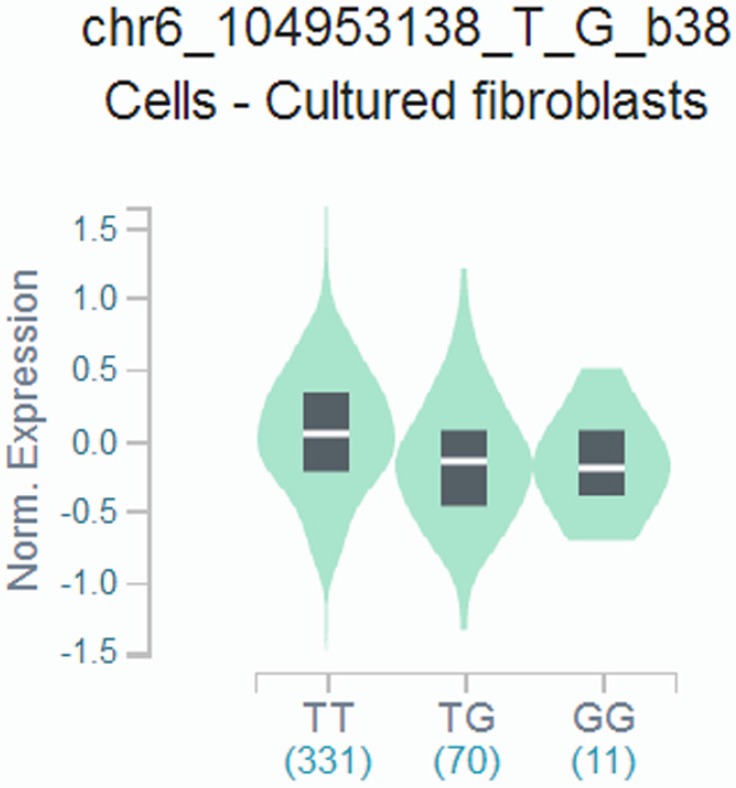
Genotype-based gene expression in transformed fibroblast cells using data from the GTEx Portal for rs9404590 T>G polymorphism (*P*=2.20*10^-4^).

We found that the rs9404590 T>G polymorphism was significantly associated with altered gene expression in transformed fibroblast cells using data from the GTEx Portal (*P*=2.20*10^-4^, **Figure 1**).

### False-positive report probability results

We preset 0.2 as the FPRP threshold. As shown in** Table 3**, at the prior probability of 0.1, the significant findings for the rs314276 C>A polymorphism remained noteworthy on the rs314276 AA genotype (FPRP=0.064), recessive model (FPRP=0.037) and the III+IV stage tumor (FPRP=0.144). All of the significant finding in rs9404590 T>G disappeared. Moreover, in the combined analysis, results with 4 risk genotypes remained noteworthy when compared with results with a risk genotype (FPRP=0.052) and 1-3 genotypes (FPRP=0.078), and in the stratified analysis, results of stage III+IV tumors also reached FPRP threshold (FPRP=0.144).

## Discussion

In this case-control study, we investigated the association between *LIN28B* SNPs with the risk of hepatoblastoma in Chinese children. To the best of our knowledge, our team is the first group to assess the association of *LIN28B* SNPs with hepatoblastoma susceptibility.

The* LIN28B* gene is located on chromosome 6q21 and encodes a miRNA-binding protein 26. The heterochronic *LIN28* gene the in *Caenorhabditis elegans* 27, and its mammalian homologue *LIN28B* gene is complementary to* lin-4* homologues miR-125 and *let-7* critical by the segment of the unusually long 3' untranslated region (UTR) in diverse mammalian tissues 17, 27-29. In this way, *LIN28B* sustains the proliferative and metabolic capacities of pluripotent stem cells and facilitates the transition of them from naive to primed pluripotency 17, 30-32. Since *LIN28B* functions as a key regulator in the diverse developing events, SNPs in the 3' UTR may play important roles. SNPs in this gene have been extensively explored concerning the regulation of secondary sexual characteristics and tumorigenesis. For example, *LIN28B* rs314276 C>A polymorphism has been shown to associate with reproductive timing 33,34, central precocious puberty 35, the linkage between puberty timing and adult disease 36, and the finger-length ratio 37. Meanwhile, this variant was also found to be associated with the risk and survival of cancers, such as Wilms tumor 22, neuroblastoma 23 and epithelial ovarian cancer 38. Previous data also showed that *LIN28B* rs221634 A>T, rs221635 T>C, and rs9404590 T>G were related to the susceptibility to Wilms tumor 22 and neuroblastoma 23.

The *LIN28B* is upregulated to repress the function of the tumor suppressor miRNA *let-7* family in the diverse tumor types and serve as a regulator of miRNA 20,39-41. The overexpression of *LIN28B* can lead to the activation of several oncogenes 42. In the previous study, miR-100, let-7a, and the *Caenorhabditis elegans lin-4* orthologue miR-125b are sharply downregulated in the hepatoblastoma 43. It is noteworthy that two of the miRNAs (miR-125b and let-7a) in the cluster are *LIN28B*-related miRNA 27, indicating a potential molecular mechanism. Meanwhile, *LIN28B* and *AURKA* expressions were upregulated in a transcriptomic and genomic analysis of human hepatoblastoma, and miRNA-26-5p inhibited hepatoblastoma by repressing *LIN28B* 44.* LIN28B* might also increase the risk of hepatoblastoma through LIN28B-RAN-AURKA pathway 44. The previous study showed that *LIN28B* is an oncofetal cancer stem cell-like marker in the recurrence of hepatocellular carcinoma 45. Hepatocellular carcinoma may have similar molecular mechanisms with hepatoblastoma, such as the activation of the Wnt/β-catenin signaling pathway 46-49. Therefore, it was reasonable to investigate the genetic implication of the *LIN28B* gene in the risk of hepatoblastoma.

Despite the findings that* LIN28B* rs94904590 T>G and rs314276 C>A could increase hepatoblastoma risk, the study suffered from some minor limitations. First, we did not find the exact molecular mechanism underlying the established associations in the study. Secondly, the sample size might be small to conclude, partly due to the low incidence rate of the disease. Validation experiments are needed in the future. Thirdly, we only recruited the Han Chinese people in our study. Fourthly, we only included four potentially functional SNPs in the *LIN28B* gene, other SNPs including the ones without functional were not included in the current study. Finally, functional experiments should be performed to further explore the role of *LIN28B* in the carcinogenesis of hepatoblastoma.

In all, our study demonstrated that *LIN28B* rs94904590 T>G and rs314276 C>A might confer increased hepatoblastoma risk in Chinese children. In the future, studies with a larger sample size are called to clarify the impact of *LIN28B* SNPs on the risk of hepatoblastoma.

## Supplementary Material

Supplementary table.

## Figures and Tables

**Table 1:** Association between *LIN28B* gene polymorphisms and hepatoblastoma susceptibility

Genotype	Cases (N=275)	Controls (N=1017)	*P*^ a^	Crude OR (95% CI)	*P*	Adjusted OR (95% CI) ^b^	*P*^ b^
**rs314276 (HWE=0.209)**							
CC	123 (44.73)	483 (47.49)		1.00		1.00	
CA	107 (38.91)	448 (44.05)		0.94 (0.70-1.25)	0.664	0.94 (0.70-1.26)	0.672
AA	45 (16.36)	86 (8.46)		**2.06 (1.36-3.10)**	**0.0006**	**2.05 (1.36-3.10)**	**0.0006**
Additive			0.017	**1.27 (1.04-1.55)**	**0.018**	**1.27 (1.04-1.55)**	**0.018**
Dominant	152 (55.27)	534 (52.51)	0.415	1.12 (0.86-1.46)	0.415	1.12 (0.86-1.46)	0.409
Recessive	230 (83.64)	931 (91.54)	0.0001	**2.12 (1.44-3.12)**	**0.0002**	**2.11 (1.43-3.12)**	**0.0002**
**rs221634 (HWE=0.969)**							
AA	94 (34.55)	342 (33.63)		1.00		1.00	
AT	144 (52.36)	495 (48.67)		1.05 (0.78-1.40)	0.758	1.04 (0.78-1.40)	0.772
TT	36 (13.09)	180 (17.70)		0.72 (0.47-1.10)	0.129	0.72 (0.47-1.10)	0.130
Additive			0.239	0.89 (0.73-1.08)	0.239	0.89 (0.73-1.08)	0.239
Dominant	180 (65.45)	675 (66.37)	0.776	0.96 (0.73-1.27)	0.775	0.96 (0.72-1.27)	0.765
Recessive	239 (86.91)	837 (82.30)	0.069	0.70 (0.48-1.03)	0.071	0.70 (0.48-1.03)	0.073
**rs221635 (HWE=0.139)**							
TT	185 (67.27)	652 (64.11)		1.00		1.00	
TC	79 (28.73)	315 (30.97)		0.88 (0.66-1.19)	0.413	0.88 (0.66-1.19)	0.407
CC	11 (4.00)	50 (4.92)		0.78 (0.40-1.52)	0.459	0.78 (0.40-1.52)	0.458
Additive			0.300	0.88 (0.70-1.12)	0.300	0.88 (0.70-1.12)	0.296
Dominant	90 (32.73)	365 (35.89)	0.330	0.87 (0.66-1.15)	0.330	0.87 (0.65-1.15)	0.325
Recessive	264 (96.00)	967 (95.08)	0.525	0.81 (0.41-1.57)	0.526	0.81 (0.41-1.57)	0.527
**rs9404590 (HWE=0.868)**							
TT	141 (51.27)	558 (54.87)		1.00		1.00	
TG	102 (37.09)	392 (38.54)		1.03 (0.77-1.37)	0.841	1.03 (0.78-1.37)	0.834
GG	32 (11.64)	67 (6.59)		**1.89 (1.19-2.99)**	**0.007**	**1.89 (1.20-3.00)**	**0.007**
Additive			0.045	**1.23 (1.00-1.51)**	**0.045**	**1.24 (1.01-1.52)**	**0.044**
Dominant	134 (48.73)	459 (45.13)	0.289	1.16 (0.89-1.51)	0.289	1.16 (0.89-1.51)	0.284
Recessive	243 (88.36)	950 (93.41)	0.005	**1.87 (1.20-2.91)**	**0.006**	**1.87 (1.20-2.92)**	**0.006**
**Combined effect of risk genotypes**							
1	44 (16.00)	219 (21.53)		1.00		1.00	
2	97 (35.27)	350 (34.41)		1.38 (0.93-2.05)	0.100	1.37 (0.93-2.04)	0.115
3	92 (33.45)	362 (35.59)		1.27 (0.85-1.88)	0.245	1.26 (0.85-1.88)	0.249
4	42 (15.27)	86 (8.46)		**2.43 (1.49-3.97)**	**0.0004**	**2.43 (1.48-3.96)**	**0.0004**
1-3	233 (84.73)	931 (91.54)		1.00		1.00	
4	42 (15.27)	86 (8.46)	0.0008	**1.95 (1.31-2.90)**	**0.0009**	**1.95 (1.31-2.90)**	**0.0009**

OR, odds ratio; CI, confidence interval; HWE, Hardy-Weinberg equilibrium. ^a^ χ^2^ test for genotype distributions between hepatoblastoma patients and controls, ^b^ Adjusted for age and gender, ^c^ Risk genotypes were carriers with rs314276 AA, rs221634 AA/AT, rs221635 TC/TT, rs9404590 TG/GG.

**Table 2:** Stratification analysis of* LIN28B* risk genotypes with hepatoblastoma susceptibility

Variables	rs314276 (cases/controls)		AOR (95% CI) ^a^	*P* ^a^	rs9404590 (cases/controls)		AOR (95% CI) ^a^	*P* ^a^	Combined (cases/controls)		AOR (95% CI) ^a^	*P* ^a^
	CC/CA	AA			TT/TG	GG			1-3	4		
Age, month												
<17	123/420	25/39	**2.19 (1.28-3.76)**	**0.005**	131/427	17/32	1.73 (0.93-3.21)	0.084	126/420	22/39	**1.88 (1.08-3.29)**	**0.027**
≥17	107/511	20/47	**2.04 (1.16-3.58)**	**0.013**	112/523	15/35	**2.02 (1.07-3.83)**	**0.031**	107/511	20/47	**2.04 (1.16-3.58)**	**0.013**
Gender												
Females	94/366	19/33	**2.21 (1.20-4.06)**	**0.011**	100/376	13/23	**2.09 (1.02-4.28)**	**0.044**	96/366	17/33	**1.94 (1.03-3.63)**	**0.039**
Males	136/565	26/53	**2.04 (1.23-3.39)**	**0.006**	143/574	19/44	1.74 (0.98-3.07)	0.057	137/565	25/53	**1.95 (1.17-3.25)**	**0.010**
Clinical stages												
I+II	124/931	18/86	1.57 (0.92-2.70)	0.101	129/950	13/67	1.44 (0.78-2.69)	0.247	125/931	17/86	1.48 (0.85-2.56)	0.168
III+IV	55/931	16/86	**3.15 (1.73-5.73)**	**0.0002**	59/950	12/67	**2.88 (1.47-5.62)**	**0.002**	55/931	16/86	**3.15 (1.73-5.73)**	**0.0002**

AOR, adjusted odds ratio; CI, confidence interval. ^a^ Adjusted for age and gender, omitting the corresponding factor.

**Table 3:** False-positive report probability analysis for significant findings

Genotype	OR (95% CI)	*P* ^a^	Statistical power ^b^	Prior probability				
				0.25	0.1	0.01	0.001	0.0001
rs314276 C>A								
AA vs. CC	2.06 (1.36-3.10)	0.0006	0.079	**0.022**	**0.064**	0.429	0.883	0.987
AA vs. CC/CA	2.12 (1.44-3.12)	0.0002	0.047	**0.013**	**0.037**	0.296	0.809	0.977
<17 month	2.19 (1.27-3.76)	0.005	0.085	**0.137**	0.324	0.840	0.982	0.998
≥17 month	2.03 (1.16-3.57)	0.014	0.147	0.217	0.454	0.902	0.989	0.999
Females	2.24 (1.22-4.12)	0.009	0.098	0.221	0.460	0.903	0.990	0.999
Males	2.04 (1.23-3.38)	0.006	0.117	**0.127**	0.304	0.828	0.980	0.998
Stage III+IV	3.15 (1.73-5.73)	0.0002	0.011	**0.053**	**0.144**	0.649	0.949	0.995
rs9404590 T>G								
GG vs. TT	1.89 (1.19-2.99)	0.007	0.177	**0.102**	0.254	0.789	0.974	0.997
GG vs. GT/TT	1.87 (1.20-2.91)	0.006	0.168	**0.095**	0.240	0.777	0.972	0.997
≥17 month	2.00 (1.06-3.79)	0.033	0.190	0.344	0.612	0.945	0.994	0.999
Females	2.13 (1.04-4.34)	0.039	0.170	0.406	0.673	0.958	0.996	1.000
Stage III+IV	2.88 (1.48-5.63)	0.002	0.032	**0.150**	0.346	0.853	0.983	0.998
Risk genotypes								
4 vs. 1	2.43 (1.49-3.97)	0.0004	0.065	**0.018**	**0.052**	0.377	0.859	0.984
4 vs. 1-3	1.95 (1.31-2.90)	0.0009	0.095	**0.028**	**0.078**	0.484	0.904	0.990
<17 month	1.88 (1.08-3.29)	0.027	0.213	0.274	0.532	0.926	0.992	0.999
≥17 month	2.03 (1.16-3.57)	0.014	0.147	0.217	0.454	0.902	0.989	0.999
Females	1.96 (1.05-3.68)	0.035	0.200	0.343	0.611	0.945	0.994	0.999
Males	1.95 (1.17-3.24)	0.011	0.160	**0.167**	0.376	0.869	0.985	0.999
Stage III+IV	3.15 (1.73-5.73)	0.0002	0.011	**0.053**	**0.144**	0.649	0.949	0.995

OR, odds ratio; CI, confidence interval. ^a^Chi-square test was used to calculate the genotype frequency distributions, ^b^Statistical power was calculated using the number of observations in each subgroup and the corresponding ORs and *P* values in this table.
