# Supplementary material for: LIN28B gene polymorphisms modify hepatoblastoma susceptibility in Chinese children
Source: J Cancer. 2020 Mar 15;11(12):3512–8. doi: 10.7150/jca.42798 (PMC7150445; doi:10.7150/jca.42798)
Supplement: Supplementary file 1 — Supplementary table. [file jcav11p3512s1.pdf]

Supplemental Table 1. Frequency distribution of selected variables in hepatoblastoma patients and controls

Variables	Cases (N=275)		Controls (N=1018)		P^{a}
	No.	%	No.	%	
Age range, month	0.10-149.97		0.033-156.00		0.365[b]
Mean ± SD	23.81 ± 25.82		25.10 ± 19.35		
<17	148	53.82	459	45.09	
≥17	127	46.18	559	54.91	
Gender					0.589
Female	113	41.09	400	39.29	
Male	162	58.91	618	60.71	
Clinical stages					
I	93	33.82			
II	49	17.82			
III	51	18.55			
IV	20	7.27			
NA	62	22.55			

SD, standard deviation, NA, not available.

[a] Two-sided χ^2 test for distributions between hepatoblastoma patients and cancer-free controls.

[b] T-test for age distribution between hepatoblastoma patients and cancer-free controls.
